# Annual Shipment of Bullion

**Published:** 1869-11

**Authors:** 


### ANNUAL SHIPMENT OF BULLION.

W. C. Child, of Wells, Fargo & Co.'s office in Treasure City, furnishes the following statement of the shipments of White Pine bullion for the year ending September 15, 1869: From Hamilton, $1,511,198.25; Treasure City, $526,891.98; Shermantown, $352,829.12; Newark, $106,238.54; Austin (White Pine bullion), $535,059.85; by other channels, $125,000. Total, $3,155,217.74. Considering that up to the month of February of this year there were not to exceed forty stamps in motion in the district, and at no time until since the last of May have we employed a greater number than sixty, the above should be accepted as a pretty fair showing. We predict that the footing up of our next annual shipment will not fall far short of $7,000,000.

With such facts as the foregoing before us, together with the steady monthly decrease of our public debt, and the continued retrenchment of Government expenses in every branch, we are not at a loss for the reason of our bonds being sought after at such high premiums, both by home and foreign capitalists, for safe and permanent investments.

With the opening of these vast fields of treasure, and the liquidation of our Government debt, together with the railways and telegraph annihilating space, and making neighbors of the citizens of New York and San Francisco within speaking distance, it is not difficult to predict a career of prosperity and peace for our country overshadowing the whole world beside.

